# Human Periodontal Ligament Stem Cell and Umbilical Vein Endothelial Cell Co-Culture to Prevascularize Scaffolds for Angiogenic and Osteogenic Tissue Engineering

**DOI:** 10.3390/ijms222212363

**Published:** 2021-11-16

**Authors:** Zeqing Zhao, Yaxi Sun, Qingchen Qiao, Li Zhang, Xianju Xie, Michael D. Weir, Abraham Schneider, Hockin H. K. Xu, Ning Zhang, Ke Zhang, Yuxing Bai

**Affiliations:** 1 Department of Orthodontics, School of Stomatology, Capital Medical University, Beijing 100050, China; zqzhao@mail.ccmu.edu.cn (Z.Z.); sunyaxi@mail.ccmu.edu.cn (Y.S.); morning@mail.ccmu.edu.cn (Q.Q.); lizhang@mail.ccmu.edu.cn (L.Z.); dentistxxj@mail.ccmu.edu.cn (X.X.); orthozhangke@mail.ccmu.edu.cn (K.Z.); 2 Biomaterials & Tissue Engineering Division, Department of Advanced Oral Sciences and Therapeutics, University of Maryland Dental School, Baltimore, MD 21201, USA; michael.weir@umaryland.edu (M.D.W.); hxu@umaryland.edu (H.H.K.X.); 3 Department of Oncology and Diagnostic Sciences, University of Maryland School of Dentistry, Baltimore, MD 21201, USA; schneider66@umaryland.edu; 4 Marlene and Stewart Greenebaum Cancer Center, University of Maryland School of Medicine, Baltimore, MD 21201, USA; 5 Center for Stem Cell Biology & Regenerative Medicine, University of Maryland School of Medicine, Baltimore, MD 21201, USA

**Keywords:** prevascularization, calcium phosphate scaffold, co-culture, human periodontal ligament stem cells, endothelial cells, bone tissue engineering

## Abstract

(1) Background: Vascularization remains a critical challenge in bone tissue engineering. The objective of this study was to prevascularize calcium phosphate cement (CPC) scaffold by co-culturing human periodontal ligament stem cells (hPDLSCs) and human umbilical vein endothelial cells (hUVECs) for the first time; (2) Methods: hPDLSCs and/or hUVECs were seeded on CPC scaffolds. Three groups were tested: (i) hUVEC group (hUVECs on CPC); (ii) hPDLSC group (hPDLSCs on CPC); (iii) co-culture group (hPDLSCs + hUVECs on CPC). Osteogenic differentiation, bone mineral synthesis, and microcapillary-like structures were evaluated; (3) Results: Angiogenic gene expressions of co-culture group were 6–9 fold those of monoculture. vWF expression of co-culture group was 3 times lower than hUVEC-monoculture group. Osteogenic expressions of co-culture group were 2–3 folds those of the hPDLSC-monoculture group. ALP activity and bone mineral synthesis of co-culture were much higher than hPDLSC-monoculture group. Co-culture group formed capillary-like structures at 14–21 days. Vessel length and junction numbers increased with time; (4) Conclusions: The hUVECs + hPDLSCs co-culture on CPC scaffold achieved excellent osteogenic and angiogenic capability in vitro for the first time, generating prevascularized networks. The hPDLSCs + hUVECs co-culture had much better osteogenesis and angiogenesis than monoculture. CPC scaffolds prevacularized via hPDLSCs + hUVECs are promising for dental, craniofacial, and orthopedic applications.

## 1. Introduction

Regeneration of large skeletal defects remains a major challenge in bone tissue engineering. The lack of vascular networks causes inadequate oxygen and nutrition supply and waste removal in the area that bone biomaterials were implanted, which compromises the efficiency of bone regeneration [1]. Recently, prevascularized bone grafts were developed to address this problem [2]. Prevascularization aims at generating a preformed microvasculature on or in scaffolds prior to their implantation [3]. Several animal studies indicated that the pre-built microcapilliary-like structures of prevascularized bone grafts eventually interconnected into the microvascular network of hosts [4].

Seeding blood vessel-forming cells onto scaffolds is the most widely used in vitro prevascularization method [3]. Vascular endothelial cells (VECs) play a critical role in angiogenesis. However, VECs alone can only form incipient vascular structures; they can not develop long-lasting stable vascular structures [5]. In contrast, co-culturing VECs and osteogenic cells (such as osteoblasts and mesenchymal stem cells (MSCs)) is a valid strategy to generate stable vascular structures [5]. Several studies indicated that the interplay between VECs and osteogenic cells is vital in the development of a functional vasculature [6]. In the process of angiogenesis, specific proangiogenic factors are crucial for VECs to migrate and form tubes [7,8,9]. MSCs can secrete vascular endothelial growth factor (VEGF), basic fibroblast growth factor (bFGF) and fibroblast growth factor (FGF) to induce the angiogenesis of VECs [7,8,9]. In return, the capillary-like structures formed by VECs were stable in the presence of MSCs due to their pericyte-like role in co-culture system [7]. MSCs can form a pericyte-like coverage around endothelial tubes, which enables the immature vessels to remain stable [7].

Calcium phosphate cement (CPC) has similar chemical characteristics to bone minerals [10]. CPC is promising for clinical applications as it has superior bioactivity and osteoconductivity [10]. In our previous studies, we prevascularized CPC scaffolds via co-seeding human bone marrow mesenchymal stem cells (hBMSCs) and human umbilical vein endothelial cells (hUVECs) [1]. hBMSCs are regarded as the gold-standard seed cell resource in bone tissue engineering. However, hBMSCs require an invasive procedure to harvest and the cell numbers are limited [11].

Human periodontal ligament stem cells (hPDLSCs) are mesenchymal stem cells (MSCs) that are isolated from the periodontal ligaments of extracted human teeth. hPDLSCs have multiple advantages. As hPDLSCs can be harvested from the extracted teeth, the patients who plan to extract their premolars for orthodontic treatments or remove the wisdom teeth can easily obtain MSCs from the extracted teeth. These patients do not need to undergo an extra invasive operation (bone marrow puncture) to obtain seed cells. As a type of MSCs, hPDLSCs have strong self-renewal ability and multipotency characteristics [11]. Compared to hBMSCs, hPDLSCs are more suitable for alveolar bone regeneration and periodontal repairs. hPDLSCs can differentiate into bone tissues as well as periodontium and cementoid tissues [12]. Furthermore, PDLSCs had vascular potential and were able to initiate in vitro angiogenesis of VECs [13]. However, to date, a literature search revealed no report on CPC scaffold prevascularization by co-culturing hPDLSCs and hUVECs.

Therefore, the objectives of this study were to: (1) co-culture hPDLSCs with hUVECs for prevascularization of CPC scaffolds for the first time; (2) compare the osteogenic effects of monocultured hPDLSCs and hPDLSCs co-cultured with hUVECs; and (3) compare the angiogenic effects of monocultured and co-cultured hPDLSCs and hUVECs. It was hypothesized that: (1) hPDLSCs co-cultured with hUVECs on CPC scaffolds could form a prevascularized construct in vitro; (2) co-cultured hPDLSCs and hUVECs have better angiogenic effects than monocultured hPDLSCs or hUVECs on CPC scaffolds; and (3) co-culturing of hPDLSCs with hUVECs could promote ALP activity and bone mineral synthesis via hPDLSCs on CPC scaffolds, compared to monoculturing hPDLSCs on CPC scaffolds.

## 2. Results

### 2.1. Identification of hPDLSCs

**Figure 1:**
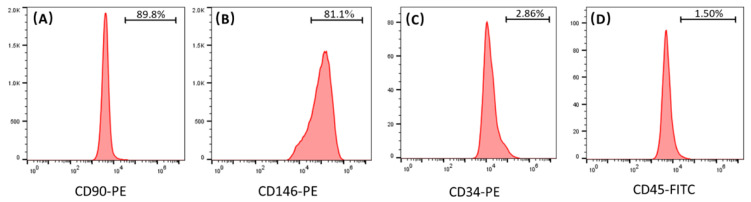
Immunophenotyping of hPDLSCs by flow cytometry. (**A**,**B**) present that the cells highly expressed MSC surface marker (CD105 and CD146). (**C**,**D**) show that cells were negative for hematopoietic markers (CD34 and CD45).

Figure 1 plots flow cytometry results of isolated hPDLSCs. The MSC surface markers CD90 was expressed to levels 89.8% in hPDLSC (Figure 1A). Another MSC surface marker, CD146, was expressed to 81.1% (Figure 1B). Figure 1C,D show that hPDLSCs exhibited a weak expression of surface markers for hematopoietic system-derived cells (CD34: 2.86% and CD45: 1.50%).

### 2.2. Evaluating Cell Proliferation via Cell Counting Kit-8 (CCK-8)

**Figure 2:**
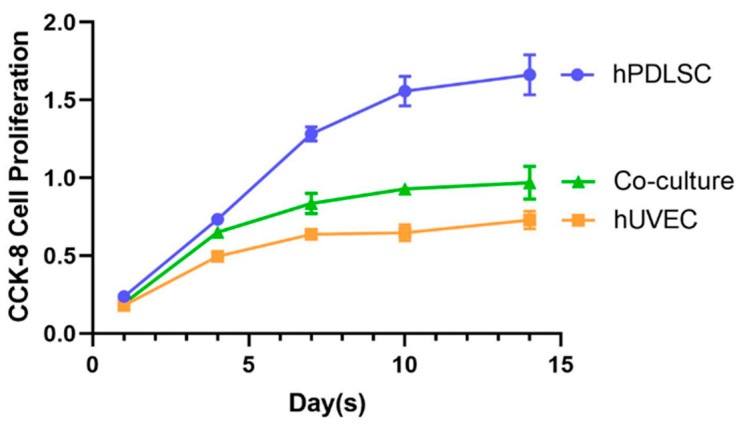
CCK-8 was used to evaluate cell proliferation at 1, 4, 7, 10, and 14 days. All groups proliferate with time, and hPDLSCs proliferated faster than hUVECs.

Figure 2 shows the results of CCK-8. The results indicate that the cell viabilities of all three groups enhanced over time. However, the proliferation rate of each group was different. The proliferation of hUVEC group increased by 3 folds from 1 day to 14 days (*p* < 0.05). Compared to hUVEC group, hPDLSC proliferated faster, with a 6-fold increase from 1 day to 14 days (*p* < 0.05). At 14 days, the cell proliferation of hPDLSC group was 2 times that of hUVEC group (*p* < 0.05). Additionally, the proliferation rate of co-culture group was between hUVEC group and hPDLSC group, with a 4-fold increase from 1 day to 14 days (*p* < 0.05). The cell activity of hPDLSC group, co-culture group, and hUVEC group were 1.663 ± 0.129, 0.970 ± 0.106 and 0.729 ± 0.057 (OD value), respectively, at 14 days.

### 2.3. Testing Angiogenic and Osteogenic Gene Expression by Quantitative Real-Time Reverse Transcription–Polymerase Chain Reaction (qRT-PCR)

**Figure 3:**
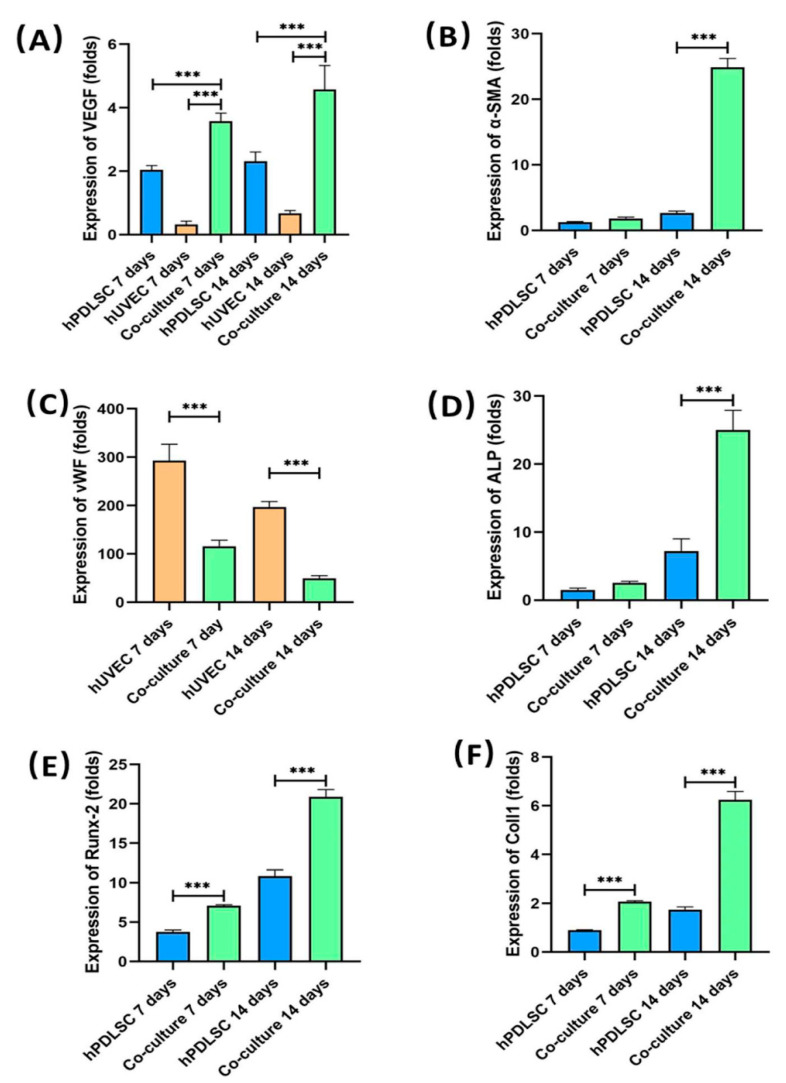
qRT-PCR assay of osteogenic and angiogenic genes at 1, 7, and 14 days: (**A**) VEGF, (**B**) α-SMA, (**C**) vWF, (**D**) ALP, (**E**) Runx-2, and (**F**) Coll-1. Co-cultured cells had higher angiogenic gene expression (VEGF and α-SMA) than monocultured hUVECs and hPDLSCs. The vWF expression of co-cultured cells was weaker than monocultured hUVECs. Additionally, the osteogenic gene expression (ALP, Runx-2 and Coll-1) of co-culture group was higher than monocultured hPDLSCs. *** *p* < 0.001.

Angiogenesis and osteogenic gene expressions are plotted in Figure 3. Figure 3A shows that, the VEGF expression of co-culture group was higher than hUVEC group and hPDLSC group at 7 days and 14 days. At 14 days, VEGF expression of co-culture group was more than 6 times that of hUVEC group (*p* < 0.05). VEGF expressions of co-culture group, hUVEC group and hPDLSC group were, respectively, 4.58 ± 0.76, 0.68 ± 0.09 and 2.32 ± 0.29 (folds) at 14 days. Figure 3B plots that α-smooth muscle actin (α-SMA) had a significantly higher expression in co-culture group than in hPDLSC group at 14 days (*p* < 0.05). At 14 days, the value of co-culture group was about 9 folds that of hPDLSC group (*p* < 0.05). The α-SMA expression of co-culture group was 24.9 ± 1.33 (folds) at 14 days. The α-SMA expression of hPDLSC group was only 2.67 ± 0.29 (folds) at 14 days. Figure 3C indicates that the expression of von Willebrand Factor (vWF) was lower in co-culture group than that in hUVEC group at 7 days and 14 days (*p* < 0.05). At 14 days, the expression of vWF in co-culture group was only 1/4 that of hUVEC group. The vWF expressions of co-culture group and hUVEC group were 49.54 ± 5.63 and 197.02 ± 11.50 (folds), respectively, at 14 days. Figure 3D–F demonstrate that the osteogenic genes of co-culture group, including alkaline phosphatase (ALP), collagen type I (Coll1) and runt-related transcription factor 2 (Runx-2), had higher expressions than hPDLSC group at 7 days and 14 days. At 14 days, the expressions of ALP, Runx-2 and Coll1 were 2–3 folds that of hPDLSC group (*p* < 0.05). For co-culture group, the expressions of ALP, Runx-2, and Coll1 were 25 ± 2.92, 20.88 ± 0.94 and 6.25 ± 0.34 (folds), respectively, at 14 days. For hPDLSC group, the expressions of ALP, Runx-2 and Coll1 were 7.21 ± 1.81, 10.85 ± 0.78 and 1.74 ± 0.11 (folds), respectively, at 14 days.

### 2.4. ALP Staining and ALP Activity

**Figure 4:**
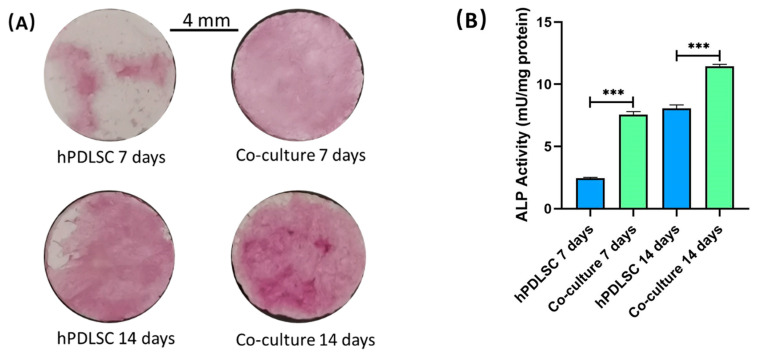
(**A**) ALP staining images of CPC scaffolds at 7 and 14 days. (**B**) ALP activity at 7 and 14 days. The images show that co-culture group has higher ALP expression than monocultured hPDLSCs. *** *p* < 0.001.

Figure 4 plots the results of ALP staining and ALP activity. As shown in Figure 4A, ALP was stained to purple. The color of hPDLSC group and co-culture group became deeper with time. Additionally, the color of co-culture group was deeper than that of co-culture group at 7 days and 14 days. Figure 4B shows that the ALP activity of co-culture group was higher that of hPDLSC group. The ALP activity of co-culture group was about 3 times that of hPDLSC at 7 days (*p* < 0.05). The ALP activity of co-culture group and hPDLSC group were (7.57 ± 0.24) mU/mg protein and (2.44 ± 0.08) mU/mg protein, respectively, at 7 days.

### 2.5. Alizarin Red Solution (ARS) Staining and Bone Mineral Quantification

**Figure 5:**
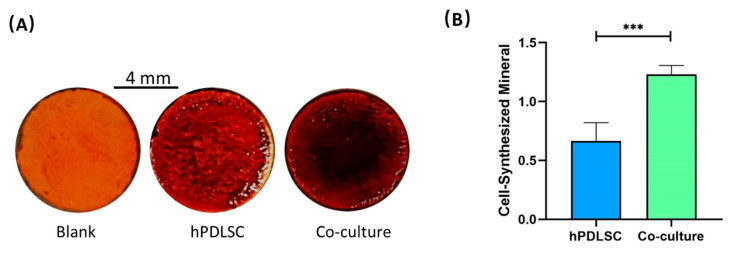
Bone mineral synthesis. (**A**) ARS staining images of CPC scaffolds at 14 days. (**B**) Quantitative assay of mineral synthesis at 14 days. Co-culture group synthesized more bone mineral compared to hPDLSC group. *** *p* < 0.001.

Figure 5 demonstrates the results of ARS staining and bone mineral quantification. The bone mineral synthesized by cells were stained red. Figure 5A shows that ARS staining was deeper and denser in co-culture group than in hPDLSC group. Additionally, it is plotted in Figure 5B that more bone mineral was synthesized in co-culture group than in hPDLSC group after culturing 14 days (*p* < 0.05). The bone mineral synthesis of co-culture group was 1.23 ± 0.08 (OD value). The bone mineral synthesis of hPDLSC group was 0.67 ± 0.15 (OD value).

### 2.6. Observing hUVECs via CD31 Immunostaining

**Figure 6:**
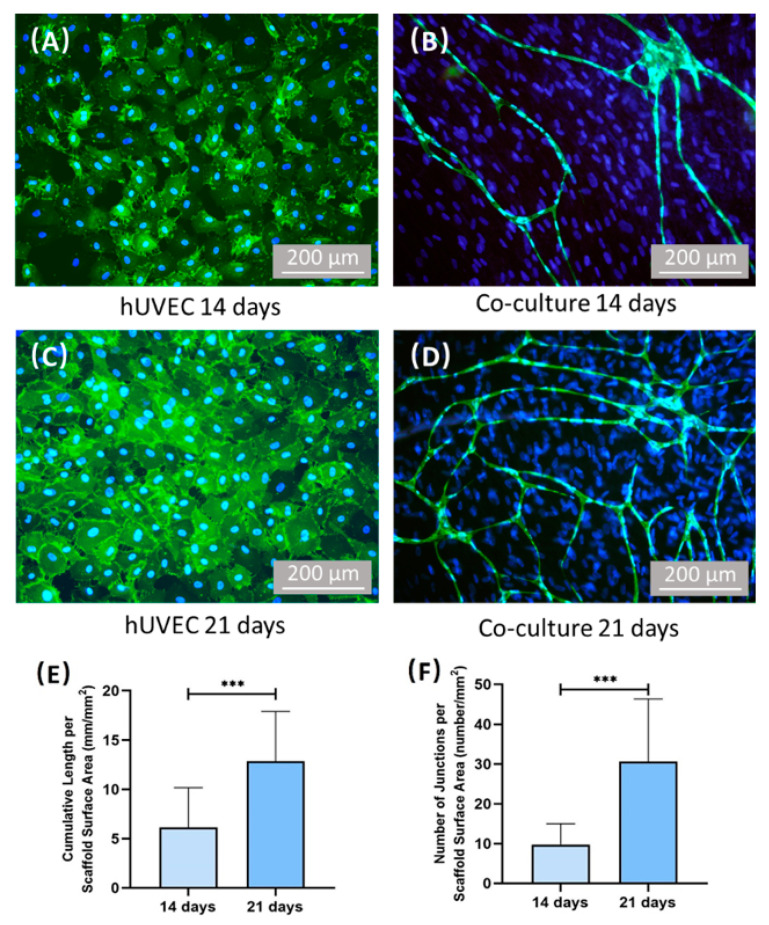
Images of CD31 immunostaining and quantification of vessel length/junctions. hUVECs were identified by immunostaining with CD31 in green on the cell membrane, and the nuclei were stained with DAPI in blue. hPDLSCs were stained by nuclei counterstaining with DAPI in blue but without green stains on the cell membrane. (**A**) hUVEC group at 14 days, (**B**) co-culture group at 14 days, (**C**) hUVEC group at 21 days, (**D**) co-culture group at 21 days, (**E**) quantification the vessel length of co-culture group, (**F**) quantification the vessel junctions of co-culture group. For hUVEC group, no capillary-like structure can be found after culturing 14 days. Co-culture group formed capillary-like structures after culturing 14 days. Additionally, the vessel length and vessel junction number of co-culture group increased with time. *** *p* < 0.001.

Figure 6 plots the images of CD31 immunostaining and the quantification of vessel length and junction number. As shown in Figure 6A–D, hUVECs were stained green with endothelial marker CD31 on the cell membrane, while cell nuclei were stained blue with DAPI. For co-culture group, more branch-like structures were observed with increasing time from 14 days to 21 days. However, for hUVEC group, no branch-like structures can be found. Figure 6E demonstrates that the accumulative vessel length of co-culture group increased with time (*p* < 0.05), and the total vessel length reached (12.88 ± 5.01) mm/mm^2^ scaffold surface area. Figure 6F shows that the junction number elevated from 14 days to 21 days (*p* < 0.05), and the final junction number reached about (30.67 ± 15.63)/mm^2^ scaffold surface area.

### 2.7. Observing the Surfaces of CPC Scaffolds by Scanning Electron Microscopy (SEM)

**Figure 7:**
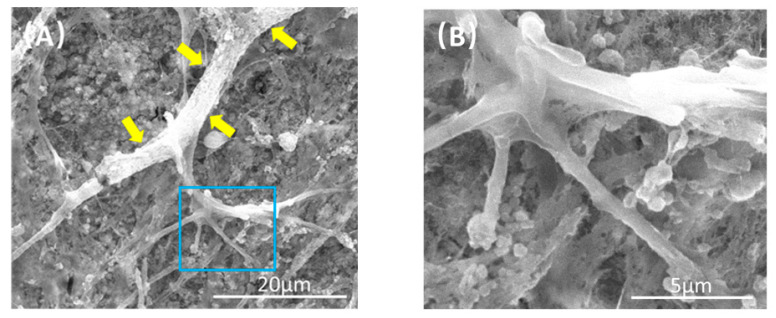
Representative SEM images of CPC scaffolds at 21 days. (**B**) is a higher magnification image of the blue dotted frame in (**A**). Capillary-like structures can be observed on the surface of CPC scaffolds (yellow arrows), and some branch-like stretches were found.

Figure 7 demonstrates the SEM pictures CPC scaffolds after culturing for 21 days. At 21 days, the CPC scaffolds of co-culture group were completely covered by cells. Additionally, microcapillary-like structures were formed on the surface of cell layers after culturing 21 days (Figure 7A). Figure 7B is a higher magnification image of the blue dotted frame in Figure 7A. It is shown in Figure 7B that some branch-like structures can be observed.

## 3. Discussion

The present study represents the first report on CPC scaffolds prevascularization by co-culturing hPDLSCs and hUVECs. The hypotheses were proven that (1) CPC scaffolds can be prevascularized by co-culturing hPDLSCs and hUVECs in vitro; (2) co-culturing with hUVECs promoted osteogenic differentiation and mineral synthesis of hPDLSCs on CPC scaffolds; and (3) co-cultured cells had better angiogenic effects than monocultured hUVECs and hPDLSCs on CPC scaffolds. Therefore, co-culturing hPDLSCs and hUVECs on CPC scaffolds is a promising strategy to enhance the bone repair effect of CPC scaffolds.

The success of bone tissue engineering during the initial phase after implantation depends on the rapid development of sufficient vascular. Prevascularization can shorten the time period during which the implanted scaffolds are avascular and suffer hypoxic conditions [3]. Thus prevascularization of bone tissue engineering constructs became a hot topic these years. Cell seeding is the most widely applied in vitro prevascularization approach [3]. In present study, co-culturing strategy was used to facilitate the prevascularization. Specific proangiogenic factors are critical for endothelial cells to migrate and form stable microcapillaries, thus mature microcapillaries cannot be generated by monoculturing hUVECs while no extra proangiogenic factors were given [14]. It was believed that MSCs can secrete proangiogenic factors which can support hUVECs to develop a functional vasculature [13]. The cellular crosstalk between osteogenic cells and endothelial cells results in the production of the required proangiogenic factors [14]. Kirkpatrick et al. proved that only a direct cell-cell contact between hUVECs and osteoblasts can form capillary-like structures [14].

In addition, MSCs played a pericyte-like role in co-culture system, it has been shown that the capillary-like structures formed by hUVECs were sustainable when MSCs were present. [7] Many authors demonstrated that MSCs were essential for stable vasculature development and co-culture is a promising way to form long-lasting microvascular networks [15]. It has been proved that co-culture of microvascular endothelial cells and osteogenic cells can successfully form microcapillary-like structures on biomaterials without further surface modification, such as coating with extracellular matrix proteins, and the survival and functioning of endothelial cells was substantially enhanced by the co-culture [16].

In the present study, hPDLSCs were used to co-culture with hUVECs. As a type of MSCs isolated from periodontal tissues, hPDLSCs have multiple characteristic advantages. Studies have indicated that PDLSCs can differentiate into periodontal tissue-forming cells, such as cementoblasts, osteoblasts, and fibroblasts [17]. Additionally, in vivo studies have demonstrated successful periodontal tissue regeneration after transplanting hPDLSCs into alveolar defects [17]. In addition, hPDLSCs reveal robust self-renewal ability which is higher than that of hBMSCs and human dental pulp stem cells [18]. Furthermore, hPDLSCs possess immunomodulatory function, which is considered to be of importance for tissue regeneration [18,19]. Therefore, hPDLSCs are considered a prime cell source for periodontal regeneration.

In addition, hPDLSCs were also thought to play important role in angiogenesis. hPDLSCs have vascular potential, they are able to produce high levels of VEGF and FGF [13]. hPDLSCs share some cell surface markers with pericytes [20]. Similar to pericytes, hPDLSCs keep themselves stay near the endothelial cell networks and help stabilize and supply nutrients to these cell networks [20]. In particular, as a type of MSCs with neural crest origin, hPDLSCs are capable of generating endothelial tissues and possess a robust angiogenic potential [21]. These characteristics make hPDLSCs an important cell population for utilization in vascularization. Thus, co-culturing hPDLSCs and hUVECs is a promising strategy to realize the prevascularization of the CPC scaffolds for bone regeneration.

In this study, the results of flow cytometry indicate that the hPDLSCs isolated from periodontal ligament tissues highly expressed CD90 and CD146, and weakly expressed CD34 and CD45. CD90 and CD146 are both MSC surface markers, and CD34 and CD45 are both hematopoietic markers. These results demonstrate that the isolated hPDLSCs indeed possessed the typical MSC characteristics. In the present study, the ratio of hPDLSCs and hUVECs was fixed at 1:3. Several studies indicated that a 1:1 ratio of MSCs and hUVECs provided robust and stable vascular networks while enabling bone-like tissue formation [7,22]. However, other studies used higher proportion of hUVECs in co-culture with MSCs, such as 3:1 and 5:1, as MSCs are larger and more proliferative [23,24]. It has been revealed that the ratio of hUVECs to MSCs will diminish with time along the co-culture experiment [24]. In the present study, CCK-8 offered a measurement of the cell viability. The results of CCK-8 show that cells in all the tested groups proliferated well on the CPC scaffolds, and the hPDLSCs proliferated faster than the hUVECs. In current study, hUVECs: hPDLSCs co-culture ratio of 3:1 may help balance the cell number with time along the experiment. However, more studies are needed to find out the best ratio of hUVECs and hPDLSCs in co-culture for angiogenesis.

VEGF, α-SMA, and vWF are all cytokines associated with angiogenesis. In the current study, the results of qRT -PCR indicate that the co-cultured cells had higher angiogenic gene expressions (VEGF and α-SMA) than the monocultured hUVECs or hPDLSCs. Furthermore, vWF had a lower expression in the co-culture group than the hUVEC group.

VEGF is the most important cytokine in promoting vascular endothelial cell proliferation and restrain apoptosis, which is crucial for the stability of vascular endothelial structure [25]. As largely reported in the literature, VEGF was proved to be a key angiogenic protein during bone repair [26]. Furthermore, VEGF is directly involved in bone remodeling and new bone formation [26]. The results of qRT-PCR show that co-cultured cells demonstrated higher VEGF gene expression than monocultured hUVECs and hPDLSCs. Crosstalk between MSCs and VECs is responsible for the proangiogenic factor expression in the co-culture [27]. Prasadam et al. reported that VEGF secreted from the osteogenic cells enhanced proliferation and migration of hUVECs and induced angiogenesis via activating VEGFR2–MAPK–ERK-signaling pathway in hUVECs [7]. If VEGF or MAPK–ERK pathways were blocked, these effects on hUVECs will be abolished [7]. In addition, it was found that interaction between MSCs and endothelial cells can upregulate angiogenic factor CXCL8 [27]. NF-κB can be activated by CXCL8 and then increased VEGF expression of co-cultured MSCs and endothelial cells [27]. Therefore, high VEGF expression in co-culture group likely contribute to its better formation of microcapillary-like structures.

vWF is a multimeric adhesive glycoprotein which synthesized and secreted by endothelial cells [28]. It is a marker of endothelial injury and dysfunction [29]. In vitro and in vivo studies have proved that lack of vWF led to enhanced vascularization [30]. The results of qRT-PCR indicated that the vWF mRNAs were expressed lower in co-culture group than in hUVEC group. The fact is consistent with the better microcapillary-like structure formation in co-culture group than in hUVEC group.

α-SMA was recognized as a pericyte marker, its modification was considered indicative of a pericyte-like differentiation of MSCs [31]. In the present study, the co-culture group expressed higher level of α-SMA than monocultured hPDLSCs. This may be due to the interaction between hUVECs and hPDLSCs. It was reported that, in co-culture system, vascular endothelial cells can communicate with MSCs via cell-cell interactions and via proximal secreted microenvironmental cues, such as growth factors and chemokines [32]. Then, MSCs will respond to these endothelial cues and undergo angiogenic differentiation [32]. Similar to pericytes, MSCs will express α-SMA, which contribute to the stable and maturation of vascular [32]. These results indicate that co-cultured hUVECs and hPDLSCs show stronger angiogenic gene expression than monocultured hUVECs and hPDLSCs.

Meanwhile, compared to monocultured hPDLSCs, the co-cultured hUVECs and hPDLSCs also demonstrated more robust osteogenic gene expression, ALP activity and mineral synthesis. Osteogenic gene expressions, ALP activity, and bone mineral synthesis are all indicators of osteogenesis. This means that the co-culture system of hUVECs and hPDLSCs can promote mineralization even in the absence of any osteogenic supplements. ALP, Runx2, and Coll1 are all important osteogenic differentiation markers of hPDLSCs [33,34,35]. The results of qRT-PCR indicated that ALP, Runx2, and Coll1 mRNAs were expressed higher in co-culture group than in hPDLSC group in present study. In addition, the results of ALP staining and ALP activity also proved that more ALP was synthesized in co-culture group than in hPDLSC group. In our previous study, co-cultured hUVECs and human osteoblasts on CPC had much higher ALP and Runx2 expressions than monocultured osteoblasts [36]. Fu et al. found that, when endothelial progenitor cells (EPC) were co-cultured with MSCs, the osteogenic differentiation of MSCs were enhanced [37]. The elevation of co-cultured cells osteogenic differentiation may be own to the paracrine communication between endothelia cells and MSCs, endothelial cells can secrete osteogenic growth factors that stimulate MSC proliferation and differentiation [37]. Indeed, an increasing number of research studies have indicated an important role of proper communication between endothelial cells and osteogenic cells in bone healing and remodeling [7]. It was demonstrated that the ALP and Coll1 expression of osteogenic cells can be upregulated by the direct contact with endothelial cells [7]. In addition, the osteogenesis of osteogenic cells can also be induced by the paracrine action of endothelial cells [7]. Additionally, the VEGF, FGF, PDGF, and TGF secreted by endothelial cells can upregulate the expression of Runx2 and ALP via activating MAPK and ERK signaling pathways [7]. Thus, the cellular crosstalk between vessel forming cell and bone-forming cells may contribute to the higher osteogenic ability of co-culture system.

CD31 is a surface marker of hUVECs. The images of CD31 immunostaining show the cell morphology of the hUVECs. The images of SEM show the microscopic morphology of the CPC scaffold surface. In the present study, monocultured hUVECs on CPC scaffolds failed to self-assemble to form microcapillary-like structures. In contrast, by immunofluorescence staining and SEM, the microvascular-like structures were observed after culturing for 14 days and 21 days in co-culture model of hUVECs and hPDLSCs. Additionally, the vessel length and junction number of microvascular-like structures elevated from 14 days to 21 days, which demonstrates that capillary network formed by co-cultured hPDLSCs and hUVECs grew with culture time. The results of immunofluorescence staining indicated that the accumulative vessel length of co-culture group was about 6 mm/mm^2^ after culturing 14 days. Interestingly, in our previous study, the density of vessels formed by co-cultured hUVECs and hBMSCs were also about 6 mm/mm^2^ after fostering 14 days [1]. Yeasmin et al. found that hPDLSCs produced appreciable levels of VEGF and FGF, and, additionally, were able to initiate in vitro angiogenesis of endothelial cells comparable to hBMSC-mediated angiogenesis [13]. This indicates that hPDLSCs could be a potential cell population for vascularization in regenerative cell therapies. Additionally, hPDLSCs are a promising alternative cell source for hBMSCs in co-culture system for vascularization.

This study represents the first report on the co-culture system of hUVECs and hPDLSCs to prevascularize CPC scaffolds. The co-cultured hPDLSCs and hUVECs demonstrated excellent angiogenic ability and formed microcapillary-like structures on CPC scaffolds. Therefore, hPDLSCs are an ideal cell source for periodontal tissue regeneration with strong angiogenic capability when co-cultured with hUVECs. The co-cultures of hPDLSCs and hUVECs were more committed to angiogenesis than the hUVECs alone. The co-cultured hUVECs promoted osteogenesis and bone mineralization of the hPDLSCs. These findings show the promise of generating a prevascularized network by co-culturing hPDLSCs and hUVECs on CPC scaffold prior to implantation to increase the vascularization and bone regeneration efficacy. The results of this study demonstrate that CPC scaffold prevascularized via co-seeding of hPDLSCs and hUVECs is a promising strategy for periodontal tissue regeneration and bone tissue engineering applications. Further studies are needed to evaluate periodontal regeneration in an animal model. Further studies are also needed to investigate the novel hPDLSCs-hUVECs-CPC prevascularization construct in other maxillofacial and orthopedic applications.

## 4. Materials and Methods

### 4.1. Harvest and Culture of hPDLSCs from Extracted Human Teeth

Periodontal ligament (PDL) tissues were collected from the roots of premolars or upper wisdom teeth that were removed from adult patients (aged 18–25 years) who had their teeth extracted due to orthodontic treatment or dental impaction. The procedures were approved by the Institutional Review Board of the University of Maryland Baltimore. All patients were informed and collected with written consent. The extracted teeth were washed with PBS contained 10% penicillin/streptomycin (Thermo Fisher Scientific, Waltham, MA, USA). Then, the PDL tissues were softly scraped from the middle third area of the root surface, and digested to cell suspension in 3 mg/mL collagenase I (Thermo Fisher Scientific, Waltham, MA, USA) with 4 mg/mL dispase (Thermo Fisher Scientific, Waltham, MA, USA) for 1 h at 37 °C in a humidified atmosphere with 5% CO_2_.

After that PDL tissues were cultured in 25 cm culture flacks (Corning, One Riverfront Plaza, Corning, NY, USA) with growth medium which consisted of Minimum Essential Medium-α medium (Biological Industries, Kibbutz Beit-Haemek, Israel) supplemented with 20% fetal bovine serum (Thermo Fisher Scientific, Waltham, MA, USA) and 1% penicillin/streptomycin, and incubated at 37 °C with 5% CO_2_. The medium was changed every three days. Cell colonies were observed about 7 days later. Every individual cell colony was digested to a single cell suspension with 0.25% Trypsin-EDTA (Thermo Fisher Scientific, Waltham, MA, USA). Then, every single cell suspension was seeded onto 24-well plates (Corning, Corning, NY, USA) and passaged to culture dishes (Corning, Corning, NY, USA) when confluency reached 80%. Cells were cultured with medium consisted of Minimum Essential Medium-α medium supplemented with 10% fetal bovine serum and 1% penicillin/streptomycin, and incubated at 37 °C with 5% CO_2_. The medium was changed on every third day.

### 4.2. Identification of Isolated hPDLSCs

The cells isolated from PDL tissues were characterized via flow cytometry. Cells (passage 2) were detached from dishes by 0.25% trypsin-EDTA and washed with phosphate-buffered saline (PBS, Thermo Fisher Scientific, Waltham, MA, USA). Then, 50 μL cell suspension that contained 1 × 10^6^ cells were incubated with the conjugated antibody against CD90-PE (Thermo Fisher Scientific, Waltham, MA, USA), CD146-PE (Thermo Fisher Scientific, Waltham, MA, USA), CD34-PE (Thermo Fisher Scientific, Waltham, MA, USA), and CD45-FITC (Thermo Fisher Scientific, Waltham, MA, USA) for 30 min on ice in the dark. Then, the cells were washed twice and resuspended with cold PBS containing 1% bovine serum albumin (BSA, Beyotime, Shanghai, China) and analyzed using a flow cytometry (Accuri C6 Flow Cytometer, BD Bioscience, San Jose, CA, USA). Data were analyzed with Flowjo software (BD Bioscience, San Jose, CA, USA). Cells between passages 3–5 were used for subsequent experiments.

### 4.3. Culture of hUVECs

Primary hUVECs were purchased from Sciencell Research Laboratories (USA). hUVECs were cultured in the endothelial cell medium (ECM, Sciencell Research Laboratories, Carlsbad, CA, USA). The medium was changed on every third day. Cells were passaged when confluency reached 80%. Passage 3–4 cells were used in present experiments.

### 4.4. Fabrication of CPC Scaffolds

CPC scaffolds were formed by powder and liquid. The CPC powder consisted tetracalcium phosphate (TTCP) and dicalcium phosphate-anhydrous (DCPA). TTCP was synthesized from a solid reaction between equimolar amounts of calcium carbonate (J.T. Baker, Avantor, Radnor, PA, USA) and DCPA (J.T. Baker, Avantor, Radnor, PA, USA) at 1500 °C for 6 h. After quenched to room temperature, ground the TTCP in a ball mill and sieved TTCP powder to obtain particles with a median particle size of 5 μm. DCPA was ground in the mechanical ball mill in 95% ethanol to obtain particles with a median sizes of 1 μm. CPC powder was obtained by blending DCPA and TTCP were at a molar ratio of 3:1. Chitosan solution was used as CPC liquid. Chitosan mixed with distilled water at a chitosan/(chitosan + water) mass fraction of 7.5% to form the CPC liquid. Blended CPC powder and liquid at a mass ratio of 3:1, then put the mixture into molds with 8 mm diameter and 1 mm thickness. Put the molds with CPC paste in the humidor for 24 h at 37 °C. After CPC scaffolds set, scaffolds were sterilized in an ethylene oxide sterilizer (Andersen, Haw River, NC, USA) for 24 h and then degassed for 3 days to remove the residual ethylene oxide.

### 4.5. Seeding and Culturing Cells on CPC Scaffolds

Three groups were tested. Group assignment is shown in Table 1.

Cultured all cell-seeded CPC scaffolds in 24-well plates. Each well contained one CPC scaffold and used 600 μL ECM medium to foster cells. Changed medium every 3 days.

### 4.6. CCK-8

CCK-8 (Dojindo, Tokyo, Japan) was used to evaluate cell proliferation at 1, 4, 7, 10, and 14 days. Five replicates in each group were used for this assay (*n* = 5). Test the cell proliferation follow the manual of CCK-8. The absorbance was measured via a microplate reader (SpectraMax Paradigm, Molecular Devices, San Jose, CA, USA) at an optical density of 450 nm.

### 4.7. qRT-PCR

qRT-PCR was performed to test the expressions of angiogenic and osteogenic genes. Collected cells at 1, 7, and 14 days. The total cellular RNA were extracted with the TRIzol reagent (Invitrogen, Thermo Fisher Scientific, Waltham, MA, USA). The concentration of RNA was measured via NanoDrop (Bio-Rad, Hercules, CA, USA). Then, the RNA was reverse transcribed into cDNA by using a cDNA reverse transcription kit (PrimeScript RT Mastermix, Takara, Shiga, Japan). TB Green Premix Ex Taq II (Takara, Shiga, Japan) was used to measure the transcript levels of the following genes: VEGF, α-SMA, vWF, ALP, Coll1, and Runx-2. The relative expression for each target gene was calculated by using the 2^−ΔΔCt^ method. The Ct values of each gene was normalized by the Ct value of glyceraldehyde 3-phosphate dehydrogenase (GAPDH). The Ct value of each group at 1 day served as the calibrator with a value of 1.

### 4.8. ALP Staining and ALP Activity

ALP staining was performed at 7 days and 14 days. BCIP/NBT Alkaline Phosphatase Color Development Kit (Beyotime, Shanghai, China) was used to stain CPC scaffolds. Five samples in each group were used for this assay (*n* = 5). Stained the CPC scaffolds follow the manual of kit.

At 7 days and 14 days, Alkaline Phosphatase Assay Kit (Beyotime, Shanghai, China) was used to test the ALP activity of all groups. Microplate reader was used to examine the ALP activity at an optical density of 450 nm. The total protein of each group was measured by BCA Protein Assay Kit (Beyotime, Shanghai, China). The concentration of protein was examined using microplate reader an optical density of 562 nm. ALP activity of each group was normalized by total protein. Each group contained five samples (*n* = 5).

### 4.9. ARS Staining and Bone Mineral Quantification

At 1, 7, and 14 days, the CPC scaffolds were fixed with 4% formaldehyde for 30 min and stained with 2% alizarin red solution (Merck, Darmstadt, Germany) for 15 min (*n* = 6). The bone mineral synthesized by cells were stained to red by alizarin red solution. Then, the alizarin red solution was removed, used PBS to softly wash the CPC scaffolds. Then, the specimens were photographed. The stained CPC scaffolds were soaked in 5% cetylpyridinium chloride (Merck, Darmstadt, Germany) for 1 h to extract the stained deposit. The amount of stained deposit was measured at optical density of 562 nm using the microplate reader. Blank CPC scaffolds (with the same treatments, but without cell seeding) were also measured. The value of blank CPC scaffolds was subtracted from the value of each group to calculate the bone mineral concentration synthesized by cells. The value of each group at 1 day was used as the calibrator with a value of 1.

### 4.10. CD31 Immunostaining

To determine the development of vascular, the samples of hUVEC group and co-culture group were stained with CD31 antibody. At 14 days and 21 days, the samples were washed with PBS, fixed with 4% parformaldehyde for 30 min, then washed three times with PBS and blocked with 0.1% bovine serum albumin (BSA, Beyotime, Shanghai, China) for 30 min. After washing three times with PBS, the samples were incubated with CD31 (PECAM-1) Mouse mAb (Cell Signaling Technology, Danvers, MA, USA) (1:500) overnight at 4 °C. The samples were then washed three times with PBS and incubated with Anti-mouse IgG (H + L) (1:1000) for 1 h. Then, wash samples with PBS for three times, and stain samples with DAPI (Beyotime, Shanghai, China) for 5 min at room temperature and washing with PBS. The samples were viewed under epifluorescent microscopy (IX73, Olympus, Tokyo, Japan). The vessels were stained green and nucleus were stained blue. Three random fields of view were imaged from each sample (five samples yielded 15 photos for each time point). The software Image J (National Institutes of Health, MD, USA) was used to measure the total length of the vessel per mm^2^. Additionally, the number of junctions per mm^2^ were also quantified.

### 4.11. SEM

After culturing 28 days. CPC scaffolds of co-culture group were examined using SEM (Quanta 200, FEI, Hillsboro, OR, USA). The samples were fixed with 4% paraformaldehyde overnight at 4 °C, dehydrated with a graded series of ethanol (25–100%), and finally rinsed with hexamethyldisilazane. The surface of CPC scaffold was sputter coated with platinum before scanning.

### 4.12. Statistical Analyses

Statistical analyses were performed using Statistical Package for the Social Sciences (SPSS 19.0, Chicago, IL, USA). All data were demonstrated as the mean value ± standard deviation. The data of CCK-8 cell activity, PCR, ALP activity and mineral synthesis were analyzed by using the one-way analysis of variance (ANOVA), followed by Tukey tests. Additionally, the data of vessel length and junction number were analyzed by using t-test. All statistical analysis was considered significant when *p* < 0.05.

## 5. Conclusions

Novel hPDLSCs-hUVECs-CPC prevascularization construct was developed for bone tissue engineering. hUVECs co-culturing with hPDLSCs on CPC scaffold achieved excellent osteogenic and angiogenic capability. The formation of microcapillary-like structures was evidenced by immunofluorescence staining and SEM examinations. Self-assembled vascular-like structures on CPC scaffolds were achieved in co-culture group, but not in monoculture groups. Furthermore, co-culturing hPDLSCs and hUVECs generated better osteogenic and angiogenic capability than monocultured hPDLSCs or hUVECs. These findings indicate the feasibility of generating prevascularized CPC scaffolds via co-culturing hPDLSCs and hUVECs, which are promising to enhance bone regeneration in a wide range of dental, craniofacial, and orthopedic applications.

## Figures and Tables

**Table 1:** Group Assignment.

**hUVEC Group**	Seed 2 × 10^5^ hUVECs on each CPC scaffold
**hPDLSC Group**	Seed 2 × 10^5^ hPDLSCs on each CPC scaffold
**Co-Culture Group**	Seed 1.5 × 10^5^ hUVECs and 5 × 10^4^ hPDLSCs on each CPC scaffold

## Data Availability

The data presented in this study are available in Supplementary Materials.

## Supplementary Materials

The following are available online at https://www.mdpi.com/article/10.3390/ijms222212363/s1.
